# NADPH: new oxygen for the ROS theory of aging

**DOI:** 10.18632/oncotarget.10744

**Published:** 2016-07-20

**Authors:** Pablo J. Fernandez-Marcos, Sandrina Nóbrega-Pereira

**Affiliations:** Bioactive Products and Metabolic Syndrome Group, Madrid Institute of Advanced Studies (IMDEA) Food, Madrid, Spain; Instituto de Medicina Molecular, Faculdade de Medicina, Universidade de Lisboa, Lisboa, Portugal

**Keywords:** aging, NADPH, G6PD, ROS, mouse

First stated in 1950 by Dentham Hannan, the free radical theory of aging hypothesized that free radicals oxidize macromolecules, creating a cumulative damage that acts as a key driver of aging. Although it enjoyed wide acceptance for many years, this proposal has been challenged by many different observations. Studies on the biological roles of reactive oxygen species (ROS) have uncovered beneficial signaling functions of these highly reactive molecules; treatment of animal models and humans with antioxidant products have failed to protect against age-induced pathologies; and overexpression of most antioxidant enzymes in animal models have not increased lifespan nor protected from aging. All these findings have led to the discredit of the free radical theory of aging in academia.

**Figure 1:**
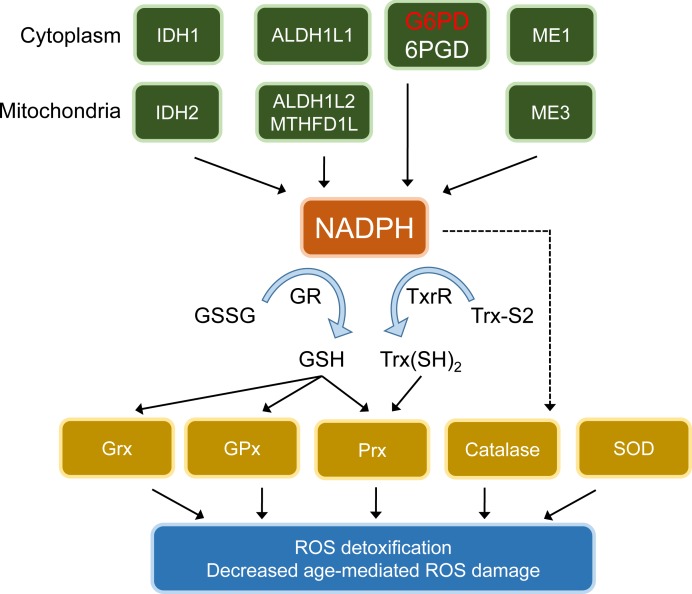
NADPH at the core of anti-ROS defenses and protection from age-induced oxidative damage NADPH can be produced by the isocitrate dehydrogenases (IDH) IDHl (cytoplasm) and IDH2 (mitochondria); the one-carbon cycle enzymes 10-formyltetrahydrofolate dehydrogenases (ALDHlL) ALDHlL1 (cytoplasm), ALDH1L2 (mitochondria) and methylenetetrahydrofolate reductase (MLHFDlL, mitochondria); the rate-limiting glucose-6-phosphate dehydrogenase (G6PD) and 6-phosphogluconate dehydrogenase (6PGD) at the oxidative branch of the pentose phosphate pathway (PPP); and the NADP-dependent malic enzymes MEl (cytoplasm) and ME3 (mitochondria). In tum, NADPH is the donor of reductive potential to glutathione reductases (GR) and thioredoxin reductases (TrxR), that reduce oxidized glutathione (GSSG) and oxidized thioredoxins (Txr-S2) to their reduced forms (GSH and Txr(SH)_2_, respectively). Glutathione peroxidases (GPx), glutaredoxins (Grx) and peroxiredoxins (Prx) are reduced by GSH, and Prx can also be reduced by Trx(SH)_2_. Catalase and superoxide dismutases (SOD) are autocatalytic enzymes that do not use NADPH as a cofactor; however, NADPH binds tightly to catalase, enhancing its activity.

A recent report by us [1] adds fresh support to the free radical theory of aging. We focused our attention on a molecule that had remained relatively unnoticed in the aging field: nicotinamide adenine dinucleotide phosphate, or NADP. Its reduced form, NADPH, is the donor of reductive potential to glutathione and thioredoxins, which in tum are used (directly or indirectly) by glutaredoxins, peroxiredoxins and glutathione peroxidases to neutralize ROS (see Figure 1). Thus, NADPH serves as the ultimate donor of reductive power for the large majority of ROS-detoxifying enzymes. NADPH can be generated by several metabolic pathways, including the reactions catalyzed by the malic enzymes, isocitrate dehydrogenases and folate dehydrogenases; but the main source of cellular NADPH are two enzymes of the oxidative branch of the pentose phosphate pathway (PPP), 6-phosphogluconate dehydrogenase (6PG) and the PPP rate-limiting enzyme glucose-6-phosphate dehydrogenase (G6PD) (see Figure 1). Importantly, overexpression of G6PD in *D. melanogaster* led to higher levels of NADPH and an increased ratio of reduced to oxidized glutathione (GSH/GSSG), concomitant with extended lifespan [2].

With this in mind, we generated a mouse line harboring a copy of the complete human G6PD gene, including exons, introns and flanking regulatory sequences: the G6PD-Tg mice. These mice displayed ubiquitous two- to four-fold overexpression of G6PD protein and mRNA and also presented an equivalent increase in G6PD activity and NADPH levels in most tissues [1]. Importantly, G6PD-Tg mice presented improved health parameters as they aged: G6PD-Tg males were more glucose tolerant and insulin sensitive; old G6PD-Tg females displayed improved neuromuscular coordination; and both male and fem ale G6PD-Tg mice tended to gain less weight with age. Moreover, G6PD- Tg females showed a 14% increase in medium lifespan. Molecularly, we observed a reduction in age-induced DNA oxidation and lipid peroxidation (the latter reduction was only significant in transgenic females). Therefore, the improved healthspan of G6PD-Tg mice can be associated to a decrease in age-associated oxidative damage to macromolecules, which is in agreement with the prediction of the free radical theory of aging.

We reasoned that an increase in NADPH would potentiate most of the anti-ROS cellular defenses, creating an overall hyper-defensive state. Of note, elevating NADPH levels should not alter the expression and subcellular localization of antioxidant enzymes. Therefore, we predicted an increase in antioxidant protection without altering the normal localization, interaction and regulation of the antioxidant proteins (see Figure). This is in contrast to previous antioxidant complementation approaches, where antioxidant molecules indiscriminately neutralize ROS. Our strategy is also different to previous genetic mouse models focused on the overexpression of a limited number of specific ROS-detoxifying enzymes, which may have partial effects or even disrupt the overall cellular antioxidant equilibrium.

Nutritional supplementation with antioxidants has been shown to promote certain human tumors [3]. Moreover, G6PD expression and/or activity are increased in a variety of cancer types [4] and NADPH production through the folate pathway can promote metastasis in a mouse model of melanoma [5]. To evaluate the potential tumorigenic risk of G6PD overexpression, we crossed G6PD-Tg mice with several tumor-prone genetically modified mice, including p53-KO (which develop sarcomas and T-cell lymphomas), ATM-KO (T-cell lymphomas), MMTV-PyMT (mammary tumors) and Efl- myc (B-cell lymphomas). In all of these combinations, mice carrying the G6PD-Tg allele presented the same tumor incidence and latency as the control mice, indicating that a moderate and regulated increase in G6PD expression and activity, or in NADPH levels, does not result in increased tumor development.

Our results lend support for the development of pharmacological or nutritional approaches aimed to increase NADPH levels as a new strategy to counteract agmg. Conceivably, this could be achieved by overactivation of NADPH-producing pathways, such as the PPP (as we have reported), the folate pathway or others. In agreement, prior reports in mice have observed beneficial cardiovascular effects by genetically increasing the PPP flux [6]. Interestingly, NADPH can also be interconverted to nicotinamide-adenine dinucleotide (NADH), and this could partially account for the increased lifespan observed in mice supplemented with nicotinamide riboside, an NADH precursor [7]. Promising future research on this field is granted.
